# Computational Information Geometry for Binary Classification of High-Dimensional Random Tensors †

**DOI:** 10.3390/e20030203

**Published:** 2018-03-17

**Authors:** Gia-Thuy Pham, Rémy Boyer, Frank Nielsen

**Affiliations:** 1 Laboratory of Signals and Systems (L2S), Department of Signals and Statistics, University of Paris-Sud, 91400 Orsay, France; giathuy.pham@l2s.centralesupelec.fr (G.-T.P.); remy.boyer@l2s.centralesupelec.fr (R.B.); 2 Computer Science Department LIX, École Polytechnique, 91120 Palaiseau, France; 3 Sony Computer Science Laboratories, Tokyo 141-0022, Japan

**Keywords:** optimal Bayesian detection, information geometry, minimal error probability, Chernoff/Bhattacharyya upper bound, large random tensor, Fisher information, large random sensing matrix

## Abstract

Evaluating the performance of Bayesian classification in a high-dimensional random tensor is a fundamental problem, usually difficult and under-studied. In this work, we consider two Signal to Noise Ratio (SNR)-based binary classification problems of interest. Under the alternative hypothesis, i.e., for a non-zero SNR, the observed signals are either a noisy rank-*R* tensor admitting a *Q*-order Canonical Polyadic Decomposition (CPD) with large factors of size $N_{q}\times R$, i.e., for 1≤q≤Q, where $R,N_{q}\to \infty$ with $R^{1/q}/N_{q}$ converge towards a finite constant or a noisy tensor admitting TucKer Decomposition (TKD) of multilinear $\left(M_{1},\ldots ,M_{Q}\right)$-rank with large factors of size $N_{q}\times M_{q}$, i.e., for 1≤q≤Q, where $N_{q},M_{q}\to \infty$ with $M_{q}/N_{q}$ converge towards a finite constant. The classification of the random entries (coefficients) of the core tensor in the CPD/TKD is hard to study since the exact derivation of the minimal Bayes’ error probability is mathematically intractable. To circumvent this difficulty, the Chernoff Upper Bound (CUB) for larger SNR and the Fisher information at low SNR are derived and studied, based on information geometry theory. The tightest CUB is reached for the value minimizing the error exponent, denoted by $s^{⋆}$. In general, due to the asymmetry of the *s*-divergence, the Bhattacharyya Upper Bound (BUB) (that is, the Chernoff Information calculated at $s^{⋆}=1/2$) cannot solve this problem effectively. As a consequence, we rely on a costly numerical optimization strategy to find $s^{⋆}$. However, thanks to powerful random matrix theory tools, a simple analytical expression of $s^{⋆}$ is provided with respect to the Signal to Noise Ratio (SNR) in the two schemes considered. This work shows that the BUB is the tightest bound at low SNRs. However, for higher SNRs, the latest property is no longer true.

## 1. Introduction

### 1.1. State-of-the-Art and Problem Statement

Evaluating the performance limit for the “Gaussian information plus noise” binary classification problem is a challenging research topic, see for instance [1,2,3,4,5,6,7]. Given a binary hypothesis problem, the Bayes’ decision rule is based on the principle of the largest posterior probability. Specifically, the Bayesian detector chooses the alternative hypothesis $H_{1}$ if $\mathrm{Pr}\left(H_{1}|y\right)>\mathrm{Pr}\left(H_{0}|y\right)$ for a given *N*-dimensional measurement vector y or the null hypothesis $H_{0}$, otherwise. Consequently, the optimal decision rule can often only be derived at the price of a costly numerical computation of the log posterior-odds ratio [3] since an exact calculation of the minimal Bayes’ error probability $P_{e}^{\left(N\right)}$ is often intractable [3,8]. To circumvent this problem, it is standard to exploit well-known bounds on $P_{e}^{\left(N\right)}$ based on information theory [9,10,11,12,13]. In particular, the Chernoff information [14,15] is asymptotically (in *N*) relied on the exponential rate of $P_{e}^{\left(N\right)}$. It turns out that the Chernoff information is very useful in many practically important problems as for instance, distributed sparse detection [16], sparse support recovery [17], energy detection [18], multi-input and multi-output (MIMO) radar processing [19,20], network secrecy [21], angular resolution limit in array processing [22], detection performance for informed communication systems [23], just to name a few. In addition, the Chernoff information bound can be tight for a minimal *s*-divergence over parameter s∈(0,1). Generally, this step requires solving numerically an optimization problem [24] and often leads to a complicated and uninformative expression of the optimal value of *s*. To circumvent this difficulty, a simplified case of s=1/2 is often used corresponding to the well-known Bhattacharyya divergence [13] at the price of a less accurate prediction of $P_{e}^{\left(N\right)}$. In information geometry, parameter *s* is often called α, and the *s*-divergence is the so-called Chernoff α-divergence [24].

The tensor decomposition theory is a timely and prominent research topic [25,26]. Confronting the problem of extracting useful information from a massive and multidimentional volume of measurements, it is shown that tensors are extremely relevant. In the standard literature, two main families of tensor decomposition are prominent, namely the Canonical Polyadic Decomposition (CPD) [26] and the Tucker decomposition (TKD)/HOSVD (High-Order SVD) [27,28]. These approaches are two possible multilinear generalization of the Singular Value Decomposition (SVD). A natural generalization to tensors of the usual concept of rank for matrices is called the CPD. The tensorial/canonical rank of a *P*-order tensor is equal to the minimal positive integer, say *R*, of unit rank tensors that must be summed up for perfect recovery. A unit rank tensor is the outer product of *P* vectors. In addition, the CPD has remarkable uniqueness properties [26] and involves only a reduced number of free parameters due to the constraint of minimality on *R*. Unfortunately, unlike the matrix case, the set of tensors with fixed (tensorial) rank is not close [29,30]. This singularity implies that the problem of the computation of the CPD is mathematically ill-posed. The consequence is that its numerical computation remains non trivial and is usually done using suboptimal iterative algorithms [31]. Note that this problem can sometimes be avoided by exploiting some natural hidden structures in the physical model [32]. The TKD [28] and the HOSVD [27] are two popular decompositions being an alternative to the CPD. Under this circumstance, alternative definition of rank is required, since the tensorial rank based on CPD scenario is no longer appropriate. In particular, stardard definition of multilinear rank defined as the set of positive integers $\left\{R_{1},\ldots ,R_{P}\right\}$ where each integer, $R_{p}$, is the usual rank of the *p*-th mode. Following the Eckart-Young theorem at each mode level [33], this construction is non-iterative, optimal and practical. In real-time computation [34] or adaptively computation [35], it is shown that this approach is suitable. However, in general, the low (multilinear) rank tensor based on this procedure is suboptimal [27]. More precisely, for tensors of order strictly greater than two, a generalization of the Eckart-Young theorem does not exist.

The classification performance of a multilinear tensor following the CPD and TKD can be derived and studied. It is interesting to note that the classification theory for tensors is very under studied. Based on our knowledge on the topic, only the publication [36] tackles this problem in the context of radar multidimensional data detection. A major difference with this publication is that their analysis is based on the performance of a low rank detection after matched filtering.

More precisely, we consider two cases where the observations are either (1) a noisy rank-*R* tensor admitting a *Q*-order CPD with large factors of size $N_{q}\times R$, i.e., for 1≤q≤Q, $R,N_{q}\to \infty$ with $R^{1/q}/N_{q}$ converging towards a finite constant, or (2) a noisy tensor admitting a TKD of multilinear $\left(M_{1},\ldots ,M_{Q}\right)$-rank with large factors of size $N_{q}\times M_{q}$, i.e., for 1≤q≤Q, where $N_{q},M_{q}\to \infty$ with $M_{q}/N_{q}$ converging towards a finite constant. A standard approach for zero-mean independent Gaussian core and noise tensors, is to define the Signal to Noise Ratio by $\mathrm{SNR}=\sigma_{s}^{2}/\sigma^{2}$ where $\sigma_{s}^{2}$ and $\sigma^{2}$ are the variances of the vectorized core and noise tensors, respectively. So, the binary classification can be described in the following way:

Under the null hypothesis $H_{0}$, SNR=0, meaning that the observed tensor contains only noise. Conversely, the alternative hypothesis $H_{1}$ is based on SNR≠0, meaning that there exists a multilinear signal of interest. First note that there exists a lack of contribution dealing with classification performance for tensors. Since the exact derivation of the error probability is intractable, the performance of the classification of the core tensor random entries is hard to evaluate. To circumvent this audible difficulty, based on computational information geometry theory, we consider the Chernoff Upper Bound (CUB), and the Fisher information in the context of massive measurement vectors. The error exponent can be minimized at $s^{⋆}$, which corresponds to the reachable tightest CUB. In general, due to the asymmetry of the *s*-divergence, the Bhattacharyya Upper Bound (BUB)—Chernoff Information calculated at $s^{⋆}=1/2$—cannot solve this problem effectively. As a consequence, we rely on a costly numerical optimization strategy to find $s^{⋆}$. However, with respect to different Signal to Noise Ratios (SNR), we provide simple analytical expressions of $s^{⋆}$, thanks to the so-called Random Matrix Theory (RMT). For low SNR, analytical expressions of the Fisher information are given. Note that the analysis of the Fisher information in the context of the RMT has been only studied in recent contributions [37,38,39] for parameter estimation. For larger SNR, analytic and simple expression of the CUB for the CPD and the TKD are provided.

We note that Random Matrix Theory (RMT) has attracted both mathematicians and physicists since they were first introduced in mathematical statistics by Wishart in 1928 [40]. When Wigner [41] introduced the concept of statistical distribution of nuclear energy levels, the subject has started to earn prominence. However, it took until 1955 before Wigner [42] introduced ensembles of random matrices. Since then, many important results in RMT were developed and analyzed, see for instance [43,44,45,46] and the references therein. In the last two decades, research on RMT has been constantly published.

Finally, let us underline that many arguments of this paper differ from the works presented in [47,48]. In [47], we tackled the problem of detection using Chernoff Upper Bound in data of type matrix in the double asymptotic regime. In [48], we established the detection problem in tensor data by analyzing the Chernoff Upper Bound. In [48], we assumed that the tensor follows the Canonical Polyadic Decomposition (CPD), we gave some analysis of Chernoff Upper Bound when the rank of the tensor is much smaller than the dimensions of the tensor. Since [47,48] are conference papers, some proofs have been omitted due to limited space. Therefore, this full paper may share the ideas in [47,48] on Information Geometry (*s*-divergence, Chernoff Upper Bound, Fisher Information, etc.), but completes [48] in a more general asymptotic regime. Moreover, in this work, we give new analysis in both scenarios (SNR small and large) whereas [48] did not, and the important and difficult new tensor scenario of the Tucker decomposition is considered. This is in our view the main difference because the CPD is a particular case of the more general decomposition of TucKer. Indeed, in the CPD, the core tensor is assumed to be diagonal.

### 1.2. Paper Organisation

The organization of the paper is as follows: In the second section, we introduce some definitions, tensor models, and the Marchenko-Pastur distribution from random matrix theory. The third section is devoted to present Chernoff Information for binary hypothesis test. The fourth section gives the main results on Fisher Information and the Chernoff bound. The numerical simulation results are given in the fifth section. We conclude our work by giving some perspectives in the Section 6. Finally, several proofs of the paper can be found in the appendix.

## 2. Algebra of Tensors and Random Matrix Theory (RMT)

In this section, we introduce some useful definitions from tensor algebra and from the spectral theory of large random matrices.

### 2.1. Multilinear Functions

#### 2.1.1. Preliminary Definitions

**Definition** **1.** *The Kronecker product of matrices X and Y of size I×J and K×N, respectively is given by*

$$\begin{array}{c} X⊗Y=\left[\begin{array}{ccc} {\left[X\right]}_{11}Y & \ldots & {\left[X\right]}_{1J}Y\\ \vdots & \vdots \\ {X]}_{I1}Y & \ldots & {\left[X\right]}_{IJ}Y \end{array}\right]\in R^{\left(IK)\times (JN\right)}. \end{array}$$

We have rank{X⊗Y}=rank{X}×rank{Y}.

**Definition** **2.** *The vectorization vec(X) of a tensor $X\in R^{M_{1}\times \ldots \times M_{Q}}$ is a vector $x\in R^{M_{1}M_{2}\ldots M_{Q}}$ defined as*

$$\begin{array}{c} x_{h}={\left[X\right]}_{m_{1},\ldots ,m_{Q}} \end{array}$$

*where $h=m_{1}+\sum_{k=2}^{Q}\left(m_{k}-1\right)M_{1}M_{2}\ldots M_{k-1}$.*

**Definition** **3.** *The q-mode product denoted by $\times_{q}$ between a tensor $X\in R^{M_{1}\times \ldots \times M_{Q}}$ and a matrix $U\in R^{K\times M_{q}}$ is denoted by $X\times_{q}U\in R^{M_{1}\times \ldots \times M_{q-1}\times K\times M_{q+1}\times \ldots \times M_{Q}}$ with*

$$\begin{array}{c} {\left[X\times_{q}U\right]}_{m_{1},\ldots ,m_{q-1},k,m_{q+1},\ldots ,m_{Q}}=\sum_{m_{q}=1}^{M_{q}}{\left[X\right]}_{m_{1},\ldots ,m_{Q}}{\left[U\right]}_{k,m_{q}} \end{array}$$

*where 1≤k≤K.*

**Definition** **4.** *The q-mode unfolding matrix of size $M_{q}\times \left(\prod_{k=1,k\neq q}^{Q}M_{k}\right)$ denoted by $X_{\left(q\right)}={\mathrm{unfold}}_{q}\left(X\right)$ of a tensor $X\in R^{M_{1}\times \ldots \times M_{q}}$ is defined according to*

$$\begin{array}{c} {\left[X_{\left(q\right)}\right]}_{M_{q},h}={\left[X\right]}_{m_{1},\ldots ,m_{Q}} \end{array}$$

*where $h=1+\sum_{k=1,k\neq q}^{Q}\left(m_{k}-1\right)\prod_{v=1,v\neq q}^{k-1}M_{v}$.*

#### 2.1.2. Canonical Polyadic Decomposition (CPD)

The rank-*R* CPD of order *Q* is defined according to

$$\begin{array}{c} X=\sum_{r=1}^{R}s_{r}\underset{X_{r}}{\underset{︸}{\left(\phi_{r}^{\left(1\right)}\circ \ldots \circ \phi_{r}^{\left(Q\right)}\right)}}\;\;with\;\;\mathrm{rank}\left\{X_{r}\right\}=1 \end{array}$$

where ○ is the outer product [25], $\phi_{r}^{\left(q\right)}\in R^{N_{q}\times 1}$ and $s_{r}$ is a real scalar.

An equivalent formulation using the *q*-mode product defined in Definition 3 is

$$X=S\times_{1}\Phi^{\left(1\right)}\times_{2}\ldots \times_{Q}\Phi^{\left(Q\right)}$$

where S is the R×…×R diagonal core tensor with ${\left[S\right]}_{r,\ldots ,r}=s_{r}$ and $\Phi^{\left(q\right)}=\left[\phi_{1}^{\left(q\right)}\ldots \phi_{R}^{\left(q\right)}\right]$ is the *q*-th factor matrix of size $N_{q}\times R$.

The *q*-mode unfolding matrix for tensor X is given by

$$X_{\left(q\right)}=\Phi^{\left(q\right)}S{\left(\Phi^{\left(Q\right)}⊙\ldots ⊙\Phi^{\left(q+1\right)}⊙\Phi^{\left(q-1\right)}⊙\ldots ⊙\Phi^{\left(1\right)}\right)}^{T}$$

where S=diag(s) with $s={\left[s_{1},\ldots ,s_{R}\right]}^{T}$ and ⊙ stands for the Khatri-Rao product [25].

#### 2.1.3. Tucker Decomposition (TKD)

The Tucker tensor model of order *Q* is defined according to

$$\begin{array}{c} X=\sum_{m_{1}=1}^{M_{1}}\sum_{m_{2}=1}^{M_{2}}\cdots \sum_{m_{Q}=1}^{M_{Q}}s_{m_{1}m_{2}\ldots m_{Q}}\left(\phi_{m_{1}}^{\left(1\right)}\circ \phi_{m_{2}}^{\left(2\right)}\circ \cdots \circ \phi_{m_{Q}}^{\left(Q\right)}\right) \end{array}$$

where $\phi_{m_{q}}^{\left(q\right)}\in R^{N_{q}\times 1}$, q=1,…,Q and $s_{m_{1}m_{2}\ldots m_{Q}}$ is a real scalar.

**Figure 1:**
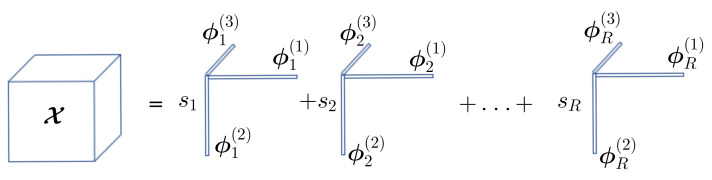
Canonical Polyadic Decomposition (CPD).

The *q*-mode product of X is similar to CPD case, however the *q*-mode unfolding matrix for tensor X is slightly different

$$\begin{array}{c} X_{\left(q\right)}=\Phi^{\left(q\right)}S_{\left(q\right)}{\left(\Phi^{\left(Q\right)}⊗\ldots ⊗\Phi^{\left(q+1\right)}⊗\Phi^{\left(q-1\right)}\ldots ⊗\Phi^{\left(1\right)}\right)}^{T} \end{array}$$

where $S_{\left(q\right)}\in R^{N_{q}\times N_{1}N_{2}\ldots N_{q-1}N_{q+1}\ldots N_{Q}}$ the *q*-mode unfolding matrix of tensor S, $\Phi^{\left(q\right)}=\left[\phi_{1}^{\left(q\right)}\ldots \phi_{M_{q}}^{\left(q\right)}\right]\in R^{N_{q}\times M_{q}}$ and ⊗ stands for Kronecker product. See Figure 1.

Following the definitions, we note that the CPD and TKD scenarios imply that vector x in Equation (11) is related either to the structured linear system $\Phi^{⊙}=\Phi^{\left(Q\right)}⊙\ldots ⊙\Phi^{\left(q+1\right)}⊙\Phi^{\left(q-1\right)}⊙\ldots ⊙\Phi^{\left(1\right)}$ or $\Phi^{⊗}=\Phi^{\left(Q\right)}⊗\ldots ⊗\Phi^{\left(q+1\right)}⊗\Phi^{\left(q-1\right)}\ldots ⊗\Phi^{\left(1\right)}$.

### 2.2. The Marchenko-Pastur Distribution

The Marchenko-Pastur distribution was introduced half a century ago [45] in 1967, and plays a key role in a number of high-dimensional signal processing problems. To help the reader, in this section, we introduce some fundamental results concerning large empirical covariance matrices. Let ${\left(v_{n}\right)}_{n=1,\ldots ,N}$ a sequence of i.i.d zero mean Gaussian random *M*-dimensional vectors for which $E\left(v_{n}v_{n}^{T}\right)=\sigma^{2}I_{M}$. We consider the empirical covariance matrix

$$\frac{1}{N}\sum_{n=1}^{N}v_{n}v_{n}^{T}$$

which can be also written as

$$\frac{1}{N}\sum_{n=1}^{N}v_{n}v_{n}^{T}=W_{N}W_{N}^{T}$$

where matrix $W_{N}$ is defined by $W_{N}=\frac{1}{\sqrt{N}}\left[v_{1},\ldots ,v_{N}\right]$. $W_{N}$ is thus a Gaussian matrix with independent identically distributed $N(0,\frac{\sigma^{2}}{N})$ entries. When N→+∞ while *M* remains fixed, matrix $W_{N}W_{N}^{T}$ converges towards $\sigma^{2}I_{M}$ in the spectral norm sense. In the high dimensional asymptotic regime defined by

$$M\to +\infty ,\;N\to +\infty ,\;c_{N}=\frac{M}{N}\to c>0$$

it is well understood that $∥W_{N}W_{N}^{T}-\sigma^{2}I_{M}∥$ does not converge towards 0. In particular, the empirical distribution ${\hat{\nu }}_{N}=\frac{1}{M}\sum_{m=1}^{M}\delta_{{\hat{\lambda }}_{m,N}}$ of the eigenvalues ${\hat{\lambda }}_{1,N}\geq \ldots \geq {\hat{\lambda }}_{M,N}$ of $W_{N}W_{N}^{T}$ does not converge towards the Dirac measure at point $\lambda =\sigma^{2}$. More precisely, we denote by $\nu_{c,\sigma^{2}}$ the Marchenko-Pastur distribution of parameters $\left(c,\sigma^{2}\right)$ defined as the probability measure

(1) $$\begin{array}{c} \nu_{c,\sigma^{2}}\left(d\lambda \right)=\delta_{0}{\left[1-\frac{1}{c}\right]}_{+}+\frac{\sqrt{\left(\lambda -\lambda^{-}\right)\left(\lambda^{+}-\lambda \right)}}{2\sigma^{2}c\pi \lambda }{⊮}_{\left[\lambda^{-},\lambda^{+}\right]\left(\lambda \right)}d\lambda \end{array}$$

with $\lambda^{-}=\sigma^{2}{\left(1-\sqrt{c}\right)}^{2}$ and $\lambda^{+}=\sigma^{2}{\left(1+\sqrt{c}\right)}^{2}$. Then, the following result holds.

**Theorem** **1**  ([45]). *The empirical eigenvalue value distribution ${\hat{\nu }}_{N}$ converges weakly almost surely towards $\nu_{c,\sigma^{2}}$ when both M and N converge towards +∞ in such a way that $c_{N}=\frac{M}{N}$ converges towards c>0. Moreover, it holds that*

(2) $$\begin{array}{cc} {\hat{\lambda }}_{1,N} & \to \sigma^{2}{\left(1+\sqrt{c}\right)}^{2}\;a.s. \end{array}$$

(3) $$\begin{array}{cc} {\hat{\lambda }}_{\min(M,N)} & \to \sigma^{2}{\left(1-\sqrt{c}\right)}^{2}\;a.s. \end{array}$$

**Figure 2:**
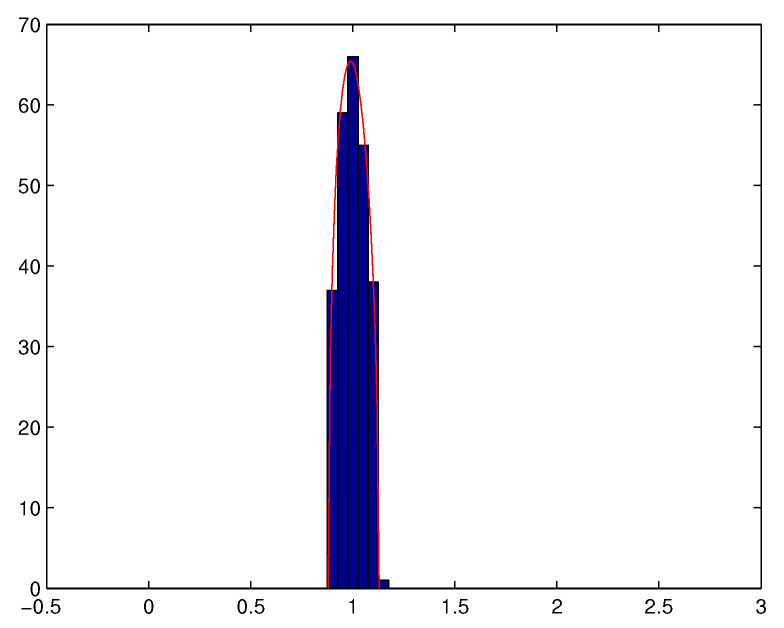
Histogram of the eigenvalues of $\frac{W_{N}W_{N}^{T}}{N}$ (with $M=256,c_{N}=\frac{M}{N}=\frac{1}{256}$, $\sigma^{2}=1$).

**Figure 3:**
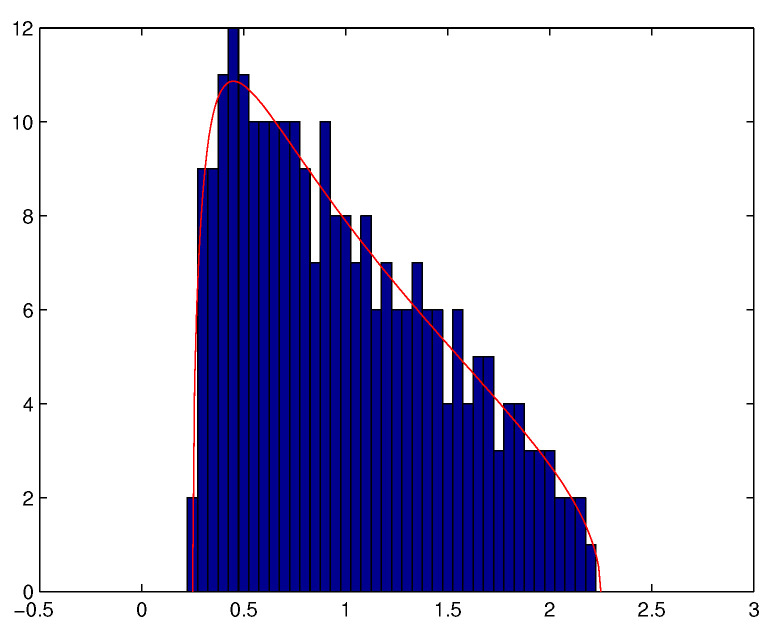
Histogram of the eigenvalues of $\frac{W_{N}W_{N}^{T}}{N}$ (with $M=256,c_{N}=\frac{M}{N}=\frac{1}{4}$, $\sigma^{2}=1$).

We also observe that Theorem 1 remains valid if $W_{N}$ is not necessarily a Gaussian matrix whose i.i.d. elements have a finite fourth order moment (see e.g., [43]). Theorem 1 means that when ratio $\frac{M}{N}$ is not small enough, the eigenvalues of the empirical spatial covariance matrix of a temporally and spatially white noise tend to spread out around the variance of the noise, and that almost surely, for *N* large enough, all the eigenvalues are located in a neighbourhood of interval $\left[\lambda^{-},\lambda^{+}\right]$. See Figure 2 and Figure 3.

## 3. Classification in a Computational Information Geometry (CIG) Framework

### 3.1. Formulation Based on a SNR-Type Criterion

We denote by $\mathrm{SNR}=\sigma_{s}^{2}/\sigma^{2}$ and $p_{i}\left(\cdot \right)=p\left(\cdot |H_{i}\right)$ with i∈{0,1}. The binary classification of the random signal based on the equi-probable binary hypothesis test, s, is

(4) $$\begin{array}{c} \left\{\begin{array}{c} H_{0}:p_{0}\left(y_{N};\Phi ,\mathrm{SNR}=0\right)=N\left(0,\Sigma_{0}\right),\\ H_{1}:p_{1}\left(y_{N};\Phi ,\mathrm{SNR}\neq 0\right)=N\left(0,\Sigma_{1}\right) \end{array}\right. \end{array}$$

where $\Sigma_{0}=\sigma^{2}I_{N}$ and $\Sigma_{1}=\sigma^{2}\left(\mathrm{SNR}\times \Phi \Phi^{T}+I_{N}\right)$. The null hypothesis data-space ($H_{0}$) is defined as $X_{0}=X\backslash X_{1}$ where

$$\begin{array}{c} X_{1}=\left\{y_{N}:\Lambda \left(y_{N}\right)=\log\frac{p_{1}\left(y_{N}\right)}{p_{0}\left(y_{N}\right)}>\tau^{\prime }\right\} \end{array}$$

is the alternative hypothesis ($H_{1}$) data-space. Following the above expression, the log-likelihood ratio test $\Lambda (y_{N})$ and the binary classification threshold $\tau^{\prime }$ are given by

$$\begin{array}{cc} \Lambda (y_{N}) & =\frac{y_{N}^{T}\Phi {\left(\Phi^{T}\Phi +\mathrm{SNR}\times I\right)}^{-1}\Phi^{T}y_{N}}{\sigma^{2}},\\ \tau^{\prime } & =-\log\det\left(\mathrm{SNR}\times \Phi \Phi^{T}+I_{N}\right) \end{array}$$

where det(·) and log(·) are respectively the determinant and the natural logarithm.

### 3.2. The Expected Log-likelihood Ratio in Geometry Perspective

We note that the estimated hypothesis $\hat{H}$ is associated to $p\left(y_{N}|\hat{H}\right)=N\left(0,\Sigma \right)$. Therefore, the expected log-likelihood ratio is defined by

$$\begin{array}{cc} E_{y_{N}|\hat{H}}\Lambda \left(y_{N}\right) & =\int_{X}p\left(y_{N}|\hat{H}\right)\log\frac{p_{1}\left(y_{N}\right)}{p_{0}\left(y_{N}\right)}dy_{N}\\ & =\mathrm{KL}(\hat{H}\left|\right|H_{0})-\mathrm{KL}(\hat{H}\left|\right|H_{1})\\ & =\frac{1}{\sigma^{2}}\mathrm{Tr}\left\{{\left(\Phi^{T}\Phi +\mathrm{SNR}\times I\right)}^{-1}\Phi^{T}\Sigma \Phi \right\} \end{array}$$

where

$$\begin{array}{c} \mathrm{KL}(\hat{H}\left|\right|H_{i})=\int_{X}p\left(y_{N}|\hat{H}\right)\log\frac{p(y_{N}|\hat{H})}{p_{i}\left(y_{N}\right)}dy_{N} \end{array}$$

is the Kullback-Leibler Divergence (KLD) [10]. The expected log-likelihood ratio test admits to a simple geometric characterization based on the difference of two KLDs [8]. However, it is often difficult to evaluate the performance of the test via the minimal Bayes’ error probability $P_{e}^{\left(N\right)}$, since its expression cannot be determined analytically in closed-form [3,8].

The minimal Bayes’ error probability conditionally to vector $y_{N}$ is defined as

$$\begin{array}{c} \mathrm{Pr}\left(\mathrm{Error}|y_{N}\right)=\frac{1}{2}\min\left\{P_{1,0},P_{0,1}\right\} \end{array}$$

where $P_{i,i^{\prime }}=\mathrm{Pr}\left(H_{i}|y_{N}\in X_{i^{\prime }}\right)$.

### 3.3. CUB

According to [24], the relation between the Chernoff Upper Bound and the (average) minimal Bayes’ error probability $P_{e}^{\left(N\right)}=E\mathrm{Pr}\left(\mathrm{Error}|y_{N}\right)$ is given by

(5) $$\begin{array}{c} P_{e}^{\left(N\right)}\leq \frac{1}{2}\times \exp\left[-{\tilde{\mu }}_{N}\left(s\right)\right] \end{array}$$

where the (Chernoff) *s*-divergence for s∈(0,1) is given by

(6) $$\begin{array}{c} {\tilde{\mu }}_{N}\left(s\right)=-\log M_{\Lambda (y_{N}|H_{1})}\left(-s\right) \end{array}$$

in which $M_{X}\left(t\right)=E\exp\left[t\times X\right]$ is the moment generating function (mgf) of variable *X*. The error exponent, denoted by $\tilde{\mu }\left(s\right)$, is given by the Chernoff information which is an asymptotic characterization on the exponentially decay of the minimal Bayes’ error probability. The error exponent is derived thanks to the Stein’s lemma according to [13]

$$\begin{array}{c} -\lim_{N\to \infty }\frac{\log P_{e}^{\left(N\right)}}{N}=\lim_{N\to \infty }\frac{{\tilde{\mu }}_{N}\left(s\right)}{N}\overset{\mathrm{def}.}{=}\tilde{\mu }\left(s\right). \end{array}$$

As parameter s∈(0,1) is free, the CUB can be tightened by minimizing this parameter:

(7) $$\begin{array}{c} s^{⋆}=\arg\max_{s\in (0,1)}\tilde{\mu }\left(s\right). \end{array}$$

Finally, using Equations (5) and (7), the Chernoff Upper Bound (CUB) is obtained. Instead of solving Equation (7), the Bhattacharyya Upper Bound (BUB) is calculated by Equation (5) and by fixing s=1/2 . Therefore we have the following relation of order:

$$\begin{array}{c} P_{e}^{\left(N\right)}\leq \frac{1}{2}\times \exp\left[-{\tilde{\mu }}_{N}\left(s^{⋆}\right)\right]\leq \frac{1}{2}\times \exp\left[-{\tilde{\mu }}_{N}\left(1/2\right)\right]. \end{array}$$

**Lemma** **1.** *The log-moment generating function given by Equation (6) for test of Equation (4) is given by*

(8) $$\begin{array}{cc} {\tilde{\mu }}_{N}\left(s\right) & =-\frac{1-s}{2}\log\det\left(\mathrm{SNR}\times \Phi \Phi^{T}+I\right)\\ & +\frac{1}{2}\log\det\left(\mathrm{SNR}\times \left(1-s\right)\Phi \Phi^{T}+I\right). \end{array}$$

**Proof.**  See Appendix A. ◻

From now on, to simplify the presentation and the numerical results later on, we denote by

$$\begin{array}{c} \mu_{N}\left(s\right)=-{\tilde{\mu }}_{N}\left(s\right)\\ \mu \left(s\right)=-\tilde{\mu }\left(s\right) \end{array}$$

for all s∈[0,1], the opposites of the log-moment generating function and its limit.

**Remark** **1.** The functions $\mu_{N}\left(s\right),\mu \left(s\right)$ are negative, since the s-divergence ${\tilde{\mu }}_{N}\left(s\right)$ is positive for all s∈[0,1].

### 3.4. Fisher Information

In the small deviation regime, we assume that δSNR is a small deviation of the SNR. The new binary hypothesis test is

$$\begin{array}{c} \left\{\begin{array}{ccc} H_{0} & : & y|\delta \mathrm{SNR}=0∼N\left(0,\Sigma (0)\right),\\ H_{1} & : & y|\delta \mathrm{SNR}\neq 0∼N\left(0,\Sigma (\delta \mathrm{SNR})\right) \end{array}\right. \end{array}$$

where $\Sigma \left(x\right)=x\times \Phi \Phi^{T}+I.$ The *s*-divergence in the small SNR deviation scenario is written as

$$\mu_{N}\left(s\right)=\frac{1-s}{2}\log\det\left[\Sigma (\delta \mathrm{SNR})\right]-\frac{1}{2}\log\det\left[\Sigma (\delta \mathrm{SNR}\times (1-s))\right]$$

**Lemma** **2.** *The s-divergence in the small deviation regime can be approximated according to*

$$\begin{array}{c} \frac{\mu_{N}\left(s\right)}{N}\overset{\delta \mathrm{SNR}\ll 1}{\approx }\left(s-1\right)s\times \frac{{\left(\delta \mathrm{SNR}\right)}^{2}}{2}\times \frac{J_{F}\left(0\right)}{N} \end{array}$$

*where the Fisher information [3] is given by*

$$\begin{array}{c} J_{F}\left(x\right)=\frac{1}{2}\mathrm{Tr}\left({\left(I+x\times \Phi \Phi^{T}\right)}^{-1}\Phi \Phi^{T}{\left(I+x\times \Phi \Phi^{T}\right)}^{-1}\Phi \Phi^{T}\right). \end{array}$$

**Proof.**  See Appendix B. ◻

According to Lemma 2, the optimal *s*-value at low SNR is $s^{⋆}\overset{\delta \mathrm{SNR}\ll 1}{=}\frac{1}{2}$. At contrary, the optimal *s*-value for larger SNR is given by the following lemma.

**Lemma** **3.** *In case of large SNR, we have*

(9) $$\begin{array}{c} s^{⋆}\overset{\mathrm{SNR}\gg 1}{\approx }1-\frac{1}{\log\mathrm{SNR}+\frac{1}{K}\sum_{n=1}^{K}\log\lambda_{n}}. \end{array}$$

*where ${\left(\lambda_{n}\right)}_{n=1,\ldots ,N}$ are the eigenvalues of $\Phi \Phi^{T}$.*

**Proof.**  See Appendix C. ◻

## 4. Computational Information Geometry for Classification

### 4.1. Formulation of the Observation Vector as a Structured Linear Model

The measurement tensor follows a noisy *Q*-order tensor of size $N_{1}\times \ldots \times N_{Q}$ can be expressed as

(10) $$\begin{array}{c} Y=X+N \end{array}$$

where N is the noise tensor whose entries are assumed to be centered i.i.d. Gaussian, i.e., ${\left[N\right]}_{n_{1},\ldots ,n_{Q}}∼N\left(0,\sigma^{2}\right)$ and the core tensor X follows either CPD or TKD given by Section 2.1.2 and Section 2.1.3, respectively. The vectorization of Equation (10) is given by

(11) $$\begin{array}{c} y_{N}=\mathrm{vec}\left(Y_{\left(1\right)}\right)=x+n \end{array}$$

where $n=\mathrm{vec}(N_{\left(1\right)})$ and $x=\mathrm{vec}(X_{\left(1\right)})$. Note that $Y_{\left(1\right)}$, $N_{\left(1\right)}$ and $X_{\left(1\right)}$ are respectively the first unfolding matrices given by Definition 4 of tensors Y,N and X,

1. When tensor X follows a *Q*-order CPD with a canonical rank of *M*, we have
  $$\begin{array}{c} x=\mathrm{vec}\left\{\Phi^{\left(1\right)}S{\left(\Phi^{\left(Q\right)}⊙\ldots ⊙\Phi^{\left(2\right)}\right)}^{T}\right\}=\Phi^{⊙}s \end{array}$$
  where $\Phi^{⊙}=\Phi^{\left(Q\right)}⊙\ldots ⊙\Phi^{\left(1\right)}$ is a N×R structured matrix and $s={\left[\begin{array}{ccc} s_{1} & \ldots & s_{R} \end{array}\right]}^{T}$ where $s_{r}∼N\left(0,\sigma_{s}^{2}\right)$, i.i.d. and $N=N_{1}\ldots N_{Q}$.
2. When tensor X follows a *Q*-order TKD of multilinear rank of $\left\{M_{1},\ldots ,M_{Q}\right\}$, we have
  $$\begin{array}{c} x=\mathrm{vec}\left\{\Phi^{\left(1\right)}S_{\left(1\right)}{\left(\Phi^{\left(Q\right)}⊗\ldots ⊗\Phi^{\left(2\right)}\right)}^{T}\right\}=\Phi^{⊗}\mathrm{vec}\left(S\right) \end{array}$$
  where $\Phi^{⊗}=\Phi^{\left(Q\right)}⊗\ldots ⊗\Phi^{\left(1\right)}$ is a N×M structured matrix with $M=M_{1}\ldots M_{Q}$ and vec(S) is the vectorization of tensor S where $s_{m_{1},\ldots ,.m_{Q}}∼N\left(0,\sigma_{s}^{2}\right)$, i.i.d.

### 4.2. The CPD Case

We recall that in the CPD case, matrix $\Phi^{⊙}=\Phi^{\left(Q\right)}⊙\ldots ⊙\Phi^{\left(1\right)}$ and ${\left(\Phi^{\left(q\right)}\right)}_{q=1,\ldots ,Q}$ are matrices of size $N_{q}\times R$. In the following, we assume that matrices $\Phi_{q=1,\ldots ,Q}^{\left(q\right)}$ are random matrices with Gaussian $N(0,\frac{1}{N_{q}})$ variate entries. We evaluate the behavior of $\frac{\mu_{N}\left(s\right)}{N}$ when ${\left(N_{q}\right)}_{q=1,\ldots ,Q}$ converge towards +∞ at the same rate and that $\frac{R}{N}$ converges towards a non zero limit.

**Result** **1.** *In the asymptotic regime where $N_{1},\ldots ,N_{Q}$ converge towards +∞ at the same rate and where R→+∞ in such a way that $c_{R}=\frac{R}{N}$ converges towards a finite constant c>0, it holds that*

(12) $$\begin{array}{cc} \frac{\mu_{N}\left(s\right)}{N} & \overset{a.s}{⟶}\mu \left(s\right)=\frac{1-s}{2}\Psi_{c}\left(\mathrm{SNR}\right)-\frac{1}{2}\Psi_{c}\left(\left(1-s\right)\times \mathrm{SNR}\right) \end{array}$$

*with a.s standing for “almost sure convergence” and*

(13) $$\begin{array}{cc} \Psi_{c}\left(x\right) & =\log\left(1+\frac{2c}{u(x)+(1-c)}\right)\\ & +c\times \log\left(1+\frac{2}{u(x)-(1-c)}\right)\\ & -\frac{4c}{x(u{\left(x\right)}^{2}-{\left(1-c\right)}^{2})} \end{array}$$

*with $u\left(x\right)=\frac{1}{x}+\sqrt{\left(\frac{1}{x}+\lambda_{c}^{+}\right)\left(\frac{1}{x}+\lambda_{c}^{-}\right)}$ where $\lambda_{c}^{\pm }={\left(1\pm \sqrt{c}\right)}^{2}$.*

**Proof.**  See Appendix D. ◻

**Remark** **2.** In [49], the Central Limit Theorem (CLT) for the linear eigenvalue statistics of the tensor version of the sample covariance matrix of type $\Phi^{⊙}{\left(\Phi^{⊙}\right)}^{T}$ is established, for $\Phi^{⊙}=\Phi^{\left(2\right)}⊙\Phi^{\left(1\right)}$, i.e., the tensor order is Q=2.

#### 4.2.1. Small SNR Deviation Scenario

In this section, we assume that SNR is small. Under this regime, we have the following result:

**Result** **2.** *In the small SNR scenario, the Fisher information for CPD is given as*

$$\begin{array}{c} \mu \left(\frac{1}{2}\right)\overset{\mathrm{SNR}\ll 1}{\approx }-\frac{{\left(\mathrm{SNR}\right)}^{2}}{16}\times c\left(1+c\right). \end{array}$$

**Proof.**  Using Lemma 2, we can notice that

$$\frac{J_{F}\left(0\right)}{N}=\frac{1}{2}\frac{R}{N}\frac{1}{R}\mathrm{Tr}\left[{\left(\Phi^{⊙}{\left(\Phi^{⊙}\right)}^{T}\right)}^{2}\right]$$

and that

$$\frac{1}{R}\mathrm{Tr}\left[{\left(\Phi^{⊙}{\left(\Phi^{⊙}\right)}^{T}\right)}^{2}\right]$$

converges a.s towards the second moment of the Marchenko-Pastur distribution which is 1+c (see for instance [43]). ◻

Note that $\mu \left(\frac{1}{2}\right)$ is the error exponent related to the Bhattacharyya divergence.

#### 4.2.2. Large SNR Deviation Scenario

**Result** **3.** *In case of large SNR, the minimizer of Chernoff Information is given by*

(14) $$\begin{array}{c} s^{⋆}\overset{\mathrm{SNR}\gg 1}{\approx }1-\frac{1}{\log\mathrm{SNR}-1-\frac{1-c}{c}\log\left(1-c\right)}. \end{array}$$

**Proof.**  It is straightforward to notice that

$$\frac{1}{K}\sum_{n=1}^{K}\log\left(\lambda_{n}\right)⟶\int_{0}^{+\infty }\log\left(\lambda \right)d\nu_{c}\left(\lambda \right)=-1-\frac{1-c}{c}\log\left(1-c\right).$$

The last equality can be obtained as in [50]. Using Lemma 3, we get immediately Equation (14). ◻

**Remark** **3.** *It is interesting to note that for c→0 or 1, the optimal s-value follows the same approximated relation given by*

$$\begin{array}{c} s^{⋆}\overset{\mathrm{SNR}\gg 1}{\approx }1-\frac{1}{\log\mathrm{SNR}} \end{array}$$

*as long as SNR≫exp[1] or equivalently a SNR in dB much larger than 4 dB.*

**Proof.**  It is straightforward to note that

$$\begin{array}{c} \frac{1-c}{c}\log\left(1-c\right)\overset{c\to 1}{⟶}0,\;and\;\frac{1-c}{c}\log\left(1-c\right)\overset{c\to 0}{⟶}-1. \end{array}$$

Using Equation (14) and condition SNR≫exp[1], the desired result is proved. ◻

#### 4.2.3. Approximated Analytical Expressions for c≪1 and Any SNR

In the case of low rank CPD where its rank *R* is supposed to be small compared to *N*, it is realistic to assume c≪1 since R≪N.

**Result** **4.** *Under this regime, the error exponent can be approximated as follows:*

$$\begin{array}{c} \mu \left(s\right)\overset{c\ll 1}{\approx }\frac{c}{2}\left((1-s)\log(1+\mathrm{SNR})-\log(1+(1-s)\mathrm{SNR})\right). \end{array}$$

**Proof.**  See Appendix E. ◻

It is easy to notice that the second-order derivative of μ(s) is strictly positive. Therefore, μ(s) is a strictly convex function over interval (0,1). As a consequence, μ(s) admits at most one global minimum. We denote by $s^{⋆}$, the global minimizer and obtained by zeroing the first-order derivative of the error exponent. This optimal value is expressed as

(15) $$\begin{array}{c} s^{⋆}\overset{c\ll 1}{\approx }1+\frac{1}{\mathrm{SNR}}-\frac{1}{\log(1+\mathrm{SNR})}. \end{array}$$

The two following scenarios can be considered:

- At low SNR, we denote by $\mu (s^{⋆})$, the error exponent associated with the tightest CUB, coincides with the error exponent associated with the BUB. To see this, when c≪1, we derive the second-order approximation of the optimal value $s^{⋆}$ in Equation (47)
  $$\begin{array}{cc} s^{⋆} & \overset{2}{\approx }1+\frac{1}{\mathrm{SNR}}\left(1-\left(1+\frac{\mathrm{SNR}}{2}\right)\right)=\frac{1}{2}. \end{array}$$
  Result 1 and the above approximation allow us to get the best error exponent at low SNR and c≪1,
  $$\begin{array}{cc} \mu \left(\frac{1}{2}\right) & \overset{\mathrm{SNR}\ll 1}{\approx }\frac{1}{4}\Psi_{c\ll 1}\left(\mathrm{SNR}\right)-\frac{1}{2}\Psi_{c\ll 1}\left(\frac{\mathrm{SNR}}{2}\right)\\ & =\frac{c}{2}\log\frac{\sqrt{1+\mathrm{SNR}}}{1+\frac{\mathrm{SNR}}{2}}. \end{array}$$
- Contrarily, when SNR→∞, $s^{⋆}\to 1$. As a consequence, the optimal error exponent in this regime is not the BUB anymore. Assuming that $\frac{\log\mathrm{SNR}}{\mathrm{SNR}}\to 0$, Equation (15) in Result 4 provides the following approximation of the optimal error exponent for large SNR
  $$\begin{array}{c} \mu \left(s^{⋆}\right)\overset{\mathrm{SNR}\gg 1}{\approx }\frac{c}{2}\left(1-\log\mathrm{SNR}+\log\log(1+\mathrm{SNR})\right). \end{array}$$

### 4.3. The TKD Case

In the TKD case, we recall that matrix $\Phi^{⊗}=\Phi^{\left(Q\right)}⊗\ldots ⊗\Phi^{\left(1\right)}$, with ${\left(\phi^{\left(q\right)}\right)}_{1\leq q\leq Q}$ are $N_{q}\times M_{q}$ dimensional matrices. We still assume that matrices $\Phi_{q=1,\ldots ,Q}^{\left(q\right)}$ are random matrices with Gaussian $N(0,\frac{1}{N_{q}})$ entries.

**Result** **5.** *In the asymptotic regime where $M_{q}<N_{q},1\leq q\leq Q$ and $M_{q},N_{q}$ converge towards +∞ at the same rate such that $\frac{M_{q}}{N_{q}}\to c_{q}$, where $0<c_{q}<1$, it holds*

$$\begin{array}{c} \frac{\mu_{N}\left(s\right)}{N}\overset{a.s}{⟶}\mu \left(s\right)=c_{1}\ldots c_{Q}\left[\frac{1-s}{2}\int_{0}^{+\infty }\ldots \int_{0}^{+\infty }\log\left(1+\mathrm{SNR}\times \lambda_{1}\ldots \lambda_{Q}\right)d\nu_{c_{1}}\left(\lambda_{1}\right)\ldots d\nu_{c_{Q}}\left(\lambda_{Q}\right)\right.\\ \;-\left.\frac{1}{2}\int_{0}^{+\infty }\ldots \int_{0}^{+\infty }\log\left(1+\left(1-s\right)\mathrm{SNR}\times \lambda_{1}\ldots \lambda_{Q}\right)d\nu_{c_{1}}\left(\lambda_{1}\right)\ldots d\nu_{c_{Q}}\left(\lambda_{Q}\right)\right] \end{array}$$

*where $\nu_{c_{q}}$ are Marchenko-Pastur distributions of parameters $\left(c_{q},1\right)$ defined as in Equation (1).*

**Proof.**  See Appendix F. ◻

**Remark** **4.** *We can notice that for Q=1, the result 5 is similar to result 1. However, when Q≥2, the integrals in Equation (16) are not tractable in a closed-form expression. For instance, let Q=2, we consider the integral*

$$\begin{array}{c} \int_{-\infty }^{+\infty }\int_{-\infty }^{+\infty }\log\left(1+\mathrm{SNR}\times \lambda_{1}\lambda_{2}\right)\nu_{c_{1}}\left(d\lambda_{1}\right)\nu_{c_{2}}\left(d\lambda_{2}\right)\\ =\int_{\lambda_{c_{1}}^{-}}^{\lambda_{c_{1}}^{+}}\int_{\lambda_{c_{2}}^{-}}^{\lambda_{c_{2}}^{+}}\log\left(1+\mathrm{SNR}\times \lambda_{1}\lambda_{2}\right)\frac{\sqrt{\left(\lambda_{1}-\lambda_{c_{1}}^{-}\right)\left(\lambda_{c_{1}}^{+}-\lambda_{1}\right)}}{2\pi c_{1}\lambda_{1}}\;\frac{\sqrt{\left(\lambda_{2}-\lambda_{c_{2}}^{-}\right)\left(\lambda_{c_{2}}^{+}-\lambda_{2}\right)}}{2\pi c_{2}\lambda_{2}}\;d\lambda_{1}\;d\lambda_{2} \end{array}$$

*where $\lambda_{c_{i}}^{\pm }={\left(1\pm \sqrt{c_{i}}\right)}^{2},i=1,2$. We can notice that this integral is characterized by elliptic integral (see e.g., [51]). As a consequence, it cannot be expressed in closed-form. However, numerical computations can be exploited to solve efficiently the minimization problem of Equation (7).*

#### 4.3.1. Large SNR Deviation Scenario

**Result** **6.** *In case of large SNR, the minimizer of Chernoff Information for TKD is given by*

$$\begin{array}{c} s^{⋆}\overset{\mathrm{SNR}\gg 1}{\approx }1-\frac{1}{\log\mathrm{SNR}-Q-\sum_{i=1}^{Q}\frac{1-c_{i}}{c_{i}}\log\left(1-c_{i}\right)}. \end{array}$$

**Proof.**  We have that

$$\begin{array}{cc} \frac{1}{M}\sum_{n=1}^{M}\log\left(\lambda_{n}\right) & ⟶\sum_{q=1}^{Q}\int_{0}^{+\infty }\log\left(\lambda_{q}\right)d\nu_{c_{q}}\left(\lambda_{q}\right)\\ & =\sum_{q=1}^{Q}\left(-1-\frac{1-c_{q}}{c_{q}}\log\left(1-c_{q}\right)\right)\\ & =-Q-\sum_{q=1}^{Q}\frac{1-c_{q}}{c_{q}}\log\left(1-c_{q}\right). \end{array}$$

Using Lemma 3, we get immediately Equation (17). ◻

#### 4.3.2. Small SNR Deviation Scenario

Under this regime, we have the following results

**Result** **7.** *For small SNR deviation, the Chernoff information for the TKD is given by*

$$\begin{array}{c} \mu \left(\frac{1}{2}\right)\overset{\delta \mathrm{SNR}\ll 1}{\approx }-\frac{{\left(\delta \mathrm{SNR}\right)}^{2}}{16}\prod_{q=1}^{Q}c_{q}\times \left(1+c_{q}\right). \end{array}$$

**Proof.**  Using Lemma 2, we can notice that

$$\frac{J_{F}\left(0\right)}{N}=\frac{1}{2}\frac{M}{N}\frac{1}{M}\mathrm{Tr}\left[{\left(\Phi^{⊗}{\left(\Phi^{⊗}\right)}^{T}\right)}^{2}\right]=\frac{1}{2}\frac{M}{N}\prod_{q=1}^{Q}\frac{\mathrm{Tr}\left[{\left(\Phi^{\left(q\right)}{\Phi^{\left(q\right)}}^{T}\right)}^{2}\right]}{M_{q}}.$$

Each term in the product converges a.s towards the second moment of Marchenko-Pastur distributions $\nu_{c_{q}}$ which are $1+c_{q}$ and $\frac{M}{N}$ converges to $\prod_{q=1}^{Q}c_{q}$. This proves the desired result. ◻

**Remark** **5.** *Contrary to the Remark 3, it is interesting to note that for $c_{1}=c_{2}=\ldots =c_{Q}=c$ and c→0 or 1, the optimal s-value follows different approximated relation given by*

$$\begin{array}{c} s^{⋆}\overset{\mathrm{SNR}\gg 1}{\underset{c\to 0}{\approx }}1-\frac{1}{\log\mathrm{SNR}} \end{array}$$

*which does not depend on Q, and*

$$\begin{array}{c} s^{⋆}\overset{\mathrm{SNR}\gg 1}{\underset{c\to 1}{\approx }}1-\frac{1}{\log\mathrm{SNR}-Q} \end{array}$$

*which depends on Q.*

In practice, when c is close to 1, we have to carefully check if Q is in the neighbourhood of log(SNR). As we can see that, when logSNR−Q<0 or 0<logSNR−Q<1, following the above approximation, $s^{⋆}\notin \left[0,1\right]$.

## 5. Numerical Illustrations

In this section, we consider cubic tensors of order Q=3 with $N_{1}=10,N_{2}=20,N_{3}=30,R=3000$ following a CPD and $M_{1}=100,M_{2}=120,M_{3}=140,N_{1}=N_{2}=N_{3}=200$ for the TKD, respectively.

**Figure 4:**
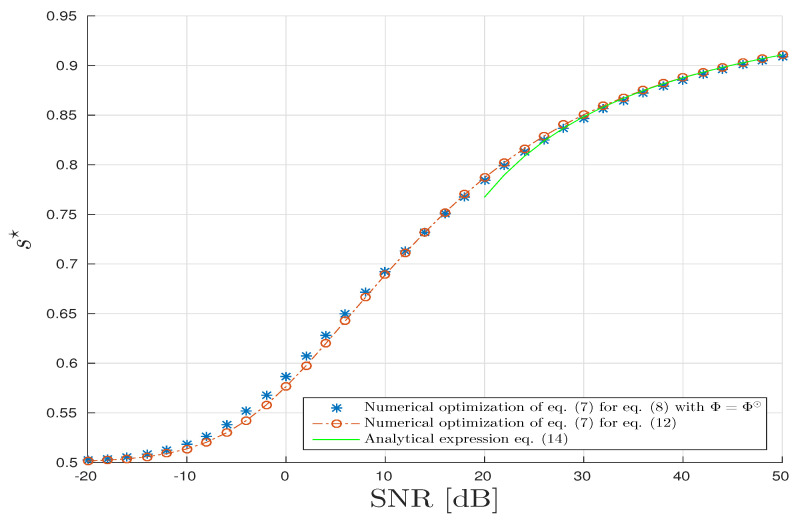
Canonical Polyadic Decomposition (CPD) scenario: Optimal *s*-parameter versus Signal to Noise Ratio (SNR) in dB.

Firstly, for the CPD model, in Figure 4, parameter $s^{⋆}$ is drawn with respect to the SNR in dB. The parameter $s^{⋆}$ is obtained thanks to three different methods. The first one is based on the brute force/exhaustive computation of the CUB by minimizing the expression in Equation (8) with $\Phi =\Phi^{⊙}$. This approach has a very high computational cost especially in our asymptotic regime (for a standard computer with Intel Xeon E5-2630 2.3 GHz and 32 GB RAM, it requires 183 h to establish 10,000 simulations). The second approach is based on the numerical optimization of the closed-form expression of μ(s) given in Result 4. In this scenario, the drawback in terms of the computational cost is largely mitigated since it consists of a minimization of a univariate regular function. Finally, under the hypothesis that SNR is large, typically >30 dB, the optimal *s*-value, $s^{⋆}$, is derived by an analytic expression given by Equation (15). We can check that the proposed semi-analytic and analytic expressions are in good agreement with the brute-force method for a lowest computational cost. Moreover, we compute the mean square relative error $\frac{1}{L}\sum_{l=1}^{L}{\left(\frac{{\hat{s}}_{l}^{⋆}-s^{⋆}}{s^{⋆}}\right)}^{2}$ where *L* = 10,000 the number of samples for Monte–Carlo process and where ${\hat{s}}_{l}^{⋆}=\arg\min_{s\in [0,1]}\mu_{N,l}\left(s\right)$ and $s^{⋆}=\arg\min_{s\in [0,1]}\mu \left(s\right)$. It turns out that the mean square relative errors are in mean of order −40 dB. We can conclude that the estimator ${\hat{s}}^{⋆}$ is a consistent estimator of $s^{⋆}$.

**Figure 5:**
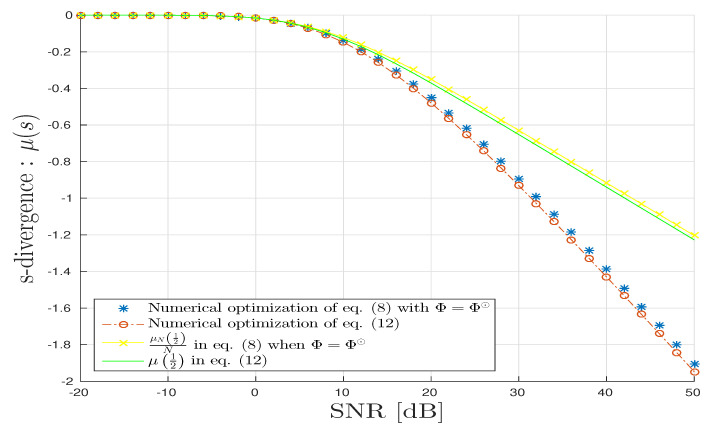
CPD scenario: *s*-divergence vs. SNR in dB.

In Figure 5, we draw various *s*-divergences: $\mu \left(\frac{1}{2}\right),\mu \left(s^{⋆}\right),\frac{1}{N}\mu_{N}\left(\frac{1}{2}\right),\frac{1}{N}\mu_{N}\left(\hat{s}\right)$. We can observe the good agreement with the proposed theoretical results. The *s*-divergence obtained by fixing $s=\frac{1}{2}$ is accurate only at small SNR but degrades when SNR grows large.

**Figure 6:**
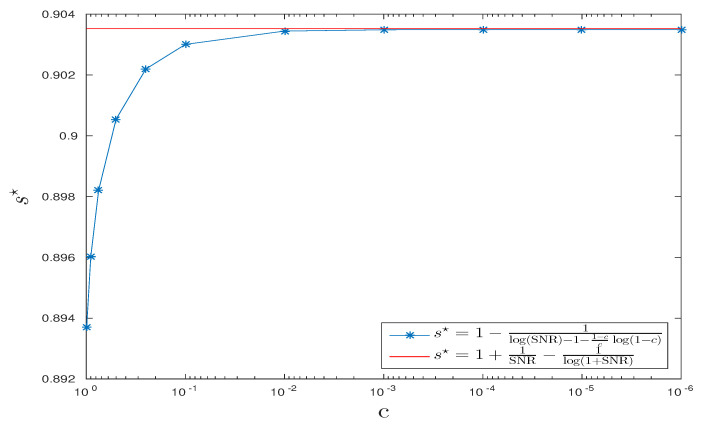
CPD scenario: $s^{⋆}$ vs. *c* , SNR=45 dB.

In Figure 6, we fix SNR=45 dB and draw $s^{⋆}$ obtained by Equation (14) versus values of $c\in \{10^{-6},10^{-5},10^{-4},10^{-3},10^{-2},10^{-1},0.25,0.5,0.75,0.9,0.99\}$ and the expression obtained by Equation (15). The two curves approach each other as *c* goes to zero as predicted by our theoretical analysis.

**Figure 7:**
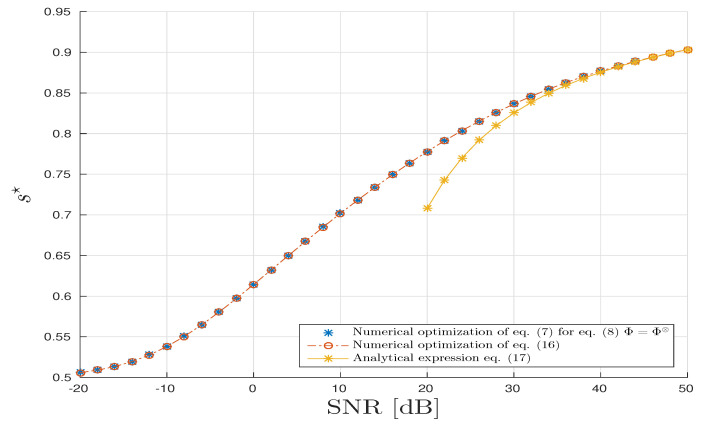
TucKer Decomposition (TKD) scenario: Optimal *s*-parameter vs. SNR in dB.

**Figure 8:**
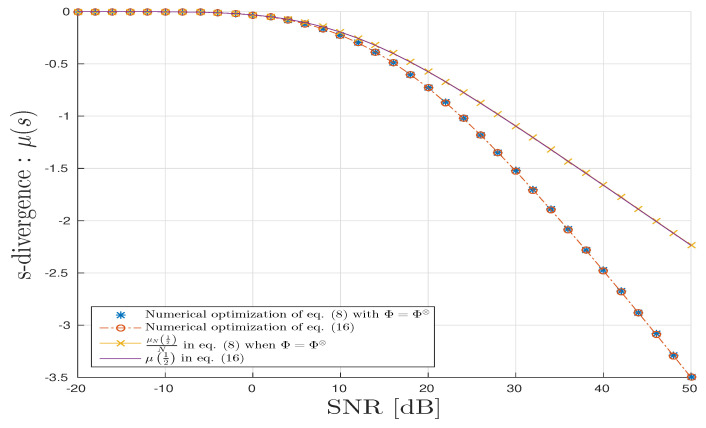
TKD scenario: *s*-divergence vs. SNR in dB.

For the TKD scenario, we follow the same methodology as above for CPD, Figure 7 and Figure 8 all agree with the analysis provided in Section 4.3.

For TKD scenario, the mean square relative error is in mean of order −40 dB. So, we check numerically the consistency of the estimator of the optimal *s*-value.

We can also notice that the convergence of $\frac{\mu_{N}\left(s\right)}{N}$ towards its deterministic equivalent μ(s) in the case TKD is faster than in the case CPD, since the dimension of matrix $\Phi^{⊗}$ is 200,200,200×100,120,140 ($N\;=\;200^{3}$) which is much larger than the dimension 6000×3000 of $\Phi^{⊙}$ (N=6000).

## 6. Conclusions

In this work, we derived and studied the limit performance in terms of minimal Bayes’ error probability for the binary classification of high-dimensional random tensors using both the tools of Information Geometry (IG) and of Random Matrix Theory (RMT). The main results on Chernoff Bounds and Fisher Information are illustrated by Monte–Carlo simulations that corroborated our theoretical analysis.

For future work, we would like to study the rate of convergence and the fluctuation of the statistics $\frac{\mu_{N}\left(s\right)}{N}$ and $\hat{s}$.

## Appendix A. Proof of Lemma 1

The *s*-divergence in Equation (6) for the following binary hypothesis test

$$\begin{array}{c} \left\{\begin{array}{ccc} H_{0} & : & y∼N\left(0,\Sigma_{0}\right),\\ H_{1} & : & y∼N\left(0,\Sigma_{1}\right) \end{array}\right. \end{array}$$

is given by [15]:

(A1) $$\begin{array}{c} {\tilde{\mu }}_{N}\left(s\right)=\frac{1}{2}\log\frac{\det(s\Sigma_{0}+\left(1-s\right)\Sigma_{1})}{{\left[\det\Sigma_{0}\right]}^{s}{\left[\det\Sigma_{1}\right]}^{1-s}}. \end{array}$$

Using the expressions of the covariance matrices $\Sigma_{0}$ and $\Sigma_{1}$, the numerator in Equation (A1) is given by

$$N\log\sigma^{2}+\log\det\left(\mathrm{SNR}\times \left(1-s\right)\Phi \Phi^{T}+I\right)$$

and the two terms at its numerator are $\log{\left[\det\Sigma_{0}\right]}^{s}=sN\log\sigma^{2}$ and

$$\log{\left[\det\Sigma_{1}\right]}^{1-s}=\left(1-s\right)\left(N\log\sigma^{2}+\log\det\left(\mathrm{SNR}\times \Phi \Phi^{T}+I\right)\right).$$

Using the above expressions, ${\tilde{mu}}_{N}\left(s\right)$ is given by Equation (8).

## Appendix B. Proof of Lemma 2

If we note $d\Sigma \left(\mathrm{SNR}\right)={\left.\frac{\partial \Sigma (x)}{\partial x}\right|}_{x=\mathrm{SNR}}$ then the following expression holds:

$$\begin{array}{c} \Sigma \left(\delta \mathrm{SNR}\right)=\Sigma \left(0\right)+\left(\delta \mathrm{SNR}\right)\times d\Sigma \left(0\right)=I+\left(\delta \mathrm{SNR}\right)\times \Phi \Phi^{T}. \end{array}$$

Using the above expression, the *s*-divergence is given by

$$\begin{array}{c} \mu_{N}\left(s\right)=\frac{1-s}{2}\log\det\left[I+\left(\delta \mathrm{SNR}\right)\times \Phi \Phi^{T}\right]-\frac{1}{2}\log\det\left[I+\delta \mathrm{SNR}\times \left(1-s\right)\times \Phi \Phi^{T}\right] \end{array}$$

Now, using Equation (8), and the following approximation:

$$\begin{array}{c} \frac{1}{N}\log\det\left(I+xA\right)=\frac{1}{N}\mathrm{Tr}\log\left(I+xA\right)\approx x\times \frac{1}{N}\mathrm{Tr}A-\frac{x^{2}}{2}\times \frac{1}{N}\mathrm{Tr}A^{2} \end{array}$$

we obtain

$$\begin{array}{c} \frac{\mu_{N}\left(s\right)}{N}\approx \left(s-1\right)s\times \frac{{\left(\delta \mathrm{SNR}\right)}^{2}}{2}\times \frac{J_{F}\left(0\right)}{N} \end{array}$$

where the Fisher information for y|δSNR∼N(0,Σ(δSNR)) is given by [3]:

$$\begin{array}{cc} J_{F}\left(\delta \mathrm{SNR}\right) & =-E\left[\frac{\partial^{2}\log p\left(y|\delta \mathrm{SNR}\right)}{\partial {\left(\delta \mathrm{SNR}\right)}^{2}}\right]\\ & =\frac{1}{2}\mathrm{Tr}\left\{\Sigma {\left(\delta \mathrm{SNR}\right)}^{-1}d\Sigma \left(\delta \mathrm{SNR}\right)\Sigma {\left(\delta \mathrm{SNR}\right)}^{-1}d\Sigma \left(\delta \mathrm{SNR}\right)\right\}\\ & =\frac{1}{2}\mathrm{Tr}{\left({\left(I+\left(\delta \mathrm{SNR}\right)\times \Phi \Phi^{T}\right)}^{-1}\Phi \Phi^{T}\left(I+\delta \mathrm{SNR}\right)\times \Phi \Phi^{T}\right)}^{-1}\Phi \Phi^{T}). \end{array}$$

## Appendix C. Proof of Theorem 3

The first step of the proof is based on the derivation of an alternative expression of $\mu_{s}\left(\mathrm{SNR}\right)$ given by Equation (A1) involving the inverse of the covariance matrices $\Sigma_{0}$ and $\Sigma_{1}$. Specifically, we have

(A2) $$\begin{array}{cc} \mu_{s}\left(\mathrm{SNR}\right) & =\frac{1}{2}\log\frac{\left(\det\Sigma_{0}\right)\left(\det\Sigma_{1}\right)\det\left(\left(1-s\right)\Sigma_{0}^{-1}+s\Sigma_{1}^{-1}\right)}{{\left[\det\Sigma_{0}\right]}^{s}{\left[\det\Sigma_{1}\right]}^{1-s}}\\ & =-\frac{1}{2}\log\frac{\det\left({\left[\left(1-s\right)\Sigma_{0}^{-1}+s\Sigma_{1}^{-1}\right]}^{-1}\right)}{{\left[\det\Sigma_{0}\right]}^{1-s}{\left[\det\Sigma_{1}\right]}^{s}}. \end{array}$$

The second step is to derive a closed-form expression in the high SNR regime using the following the approximation (see [52] for instance): ${\left(x\times \Phi \Phi^{T}+I\right)}^{-1}\overset{x\gg 1}{\approx }\Pi_{\Phi }^{⊥}=I_{N}-\Phi \Phi^{†}$ where $\Pi_{\Phi }^{⊥}$ is an orthogonal projector such as $\Pi_{\Phi }^{⊥}\Phi =0$ and $\Phi^{†}={\left(\Phi^{T}\Phi \right)}^{-1}\Phi^{T}$. The numerator in Equation (A2) is given by

$$\begin{array}{cc} {\left[\left(1-s\right)\Sigma_{0}^{-1}+s\Sigma_{1}^{-1}\right]}^{-1} & \overset{\mathrm{SNR}\gg 1}{\approx }\sigma^{2}{\left(I_{N}-sI_{N}+s\Pi_{\Phi }^{⊥}\right)}^{-1}\\ & =\sigma^{2}{\left(I_{N}-s\Phi \Phi^{†}\right)}^{-1}. \end{array}$$

As $s\Phi \Phi^{†}$ is a rank-*K* projector matrix scaled by factor s>0, its eigen-spectrum is given by $\left\{{\underset{︸}{s,\ldots ,s}}_{K},{\underset{︸}{0,\ldots ,0}}_{N-K}\right\}$. In addition, as the rank-*N* identity matrix and the scaled projector $s\Phi \Phi^{†}$ can be diagonalized in the same orthonormal basis matrix, the *n*-th eigenvalue of the inverse of matrix $I_{N}-s\Phi \Phi^{†}$ is given by

$$\begin{array}{cc} \lambda_{n}\left\{{\left(I_{N}-s\Phi \Phi^{†}\right)}^{-1}\right\} & =\frac{1}{\lambda_{n}\left\{I_{N}\right\}-s\lambda_{n}\left\{\Phi \Phi^{†}\right\}}\\ & =\left\{\begin{array}{cc} \frac{1}{1-s}, & 1\leq n\leq K,\\ 1, & K+1\leq n\leq N \end{array}\right. \end{array}$$

with s∈(0,1). Using the above property, we obtain

$$\begin{array}{cc} \log\det\left({\left[I_{N}-s\Phi \Phi^{†}\right]}^{-1}\right) & =\log\prod_{n=1}^{N}\lambda_{n}\left\{{\left(I_{N}-s\Phi \Phi^{†}\right)}^{-1}\right\}\\ & =-K\log(1-s). \end{array}$$

In addition, we have

$$\begin{array}{c} \log\det\left(\mathrm{SNR}\times \Phi \Phi^{T}+I\right)\overset{\mathrm{SNR}\gg 1}{\approx }\mathrm{Tr}\log\left(\mathrm{SNR}\times \Phi^{T}\Phi \right)=K\times \log\mathrm{SNR}+\sum_{n=1}^{K}\log\lambda_{n} \end{array}$$

Finally, thanks to Equation (A2), we have

$$\begin{array}{cc} \frac{\mu_{s}\left(\mathrm{SNR}\right)}{N} & \overset{\mathrm{SNR}\gg 1}{\approx }\frac{1}{2}\frac{K}{N}\left(\log\left(1-s\right)+s\times \log\mathrm{SNR}+\frac{s}{K}\sum_{n=1}^{K}\log\lambda_{n}\right) \end{array}$$

Finally, to obtain $s^{⋆}$ in Equation (9), we solve $\frac{\partial \mu_{s}\left(\mathrm{SNR}\right)}{\partial s}=0$.

## Appendix D. Proof of Result 1

The asymptotic behavior of $\frac{\mu_{N}\left(s\right)}{N}$ when $N_{q}\to +\infty$ for each q=1,…,Q, R→+∞ in such a way that $\frac{R^{1/q}}{N_{q}}$ converge towards a non zero constant for each q=1,…,Q can be obtained thanks to large random matrix theory. We suppose that $N_{1},\ldots ,N_{Q}$ converge towards +∞ at the same rate (i.e., $\frac{N_{q}}{N_{p}}$ converge towards a non zero constant for each (p,q)), and $c_{R}=\frac{R}{N}$ converges towards a constant c>0. Under this regime, the empirical eigenvalue distribution of covariance matrix $\Phi^{⊙}{\left(\Phi^{⊙}\right)}^{T}$ is known to converge towards the so-called Marcenko–Pastur distribution. By Section 2.2, we recall that the Marcenko–Pastur distribution $\nu_{c}\left(d\lambda \right)$ is defined as

$$\nu_{c}\left(d\lambda \right)=\delta \left(\lambda \right)\;{\left[1-c\right]}_{+}+\frac{\sqrt{\left(\lambda -\lambda_{c}^{-}\right)\left(\lambda_{c}^{+}-\lambda \right)}}{2\pi \lambda }{⊮}_{\left[\lambda_{c}^{-},\lambda_{c}^{+}\right]}\left(\lambda \right)\;d\lambda$$

where $\lambda_{c}^{-}={\left(1-\sqrt{c}\right)}^{2}$ and $\lambda_{c}^{+}={\left(1+\sqrt{c}\right)}^{2}$. We define $t_{c}\left(z\right)=\int_{R^{+}}\frac{\nu_{c}\left(d\lambda \right)}{\lambda -z}$ the Stieltjes transform of $\nu_{c}$. We have that $t_{c}\left(z\right)$ satisfies the equation

$$t_{c}\left(z\right)={\left[-z+\frac{c}{1+t_{c}\left(z\right)}\right]}^{-1}.$$

When $z\in R^{-*}$, i.e., z=−ρ, with ρ>0, it is well known that $t_{c}\left(\rho \right)$ is given by

(A3) $$t_{c}\left(-\rho \right)=\frac{2}{\rho -\left(1-c\right)+\sqrt{\left(\rho +\lambda_{c}^{-}\right)\left(\rho +\lambda_{c}^{+}\right)}}$$

It was established for the first time in [45] that if X represents a K×P random matrix with zero mean and $\frac{1}{K}$ variance i.i.d. entries, and if ${\left(\lambda_{k}\right)}_{k=1,\ldots ,K}$ represent the eigenvalues of $XX^{T}$ arranged in decreasing order, then $\frac{1}{K}\sum_{k=1}^{K}\delta \left(\lambda -\lambda_{k}\right)$, the empirical eigenvalue distribution of $XX^{T}$ converges weakly almost surely towards $\nu_{c}$, under the regime K→+∞, P→+∞, $\frac{P}{K}\to c$. In addition, we have the following property, for each continuous function f(λ)

(A4) $$\frac{1}{K}\sum_{k=1}^{K}f\left(\lambda_{k}\right)\overset{a.s}{⟶}\int_{R^{+}}f\left(\lambda \right)\;\nu_{c}\left(d\lambda \right).$$

Practically, when *K* and *P* are large enough, the histogram of the eigenvalues of each realization of $XX^{T}$ accumulates around the graph of the probability density of $\nu_{c}$.

The columns ${\left(\phi_{r}\right)}_{r=1,\ldots ,R}$ of $\Phi^{⊙}$ are vectors ${\left(\phi_{r}^{\left(Q\right)}⊗\ldots ⊗\phi_{r}^{\left(1\right)}\right)}_{r=1,\ldots ,R}$, which are mutually independent, identically distributed, and satisfy $E\left(\phi_{r}\phi_{r}^{T}\right)=\frac{I_{N}}{N}$. However, since the components of each column $\phi_{r}$ are not independent, it results in that the entries of $\Phi^{⊙}$ are not mutually independent. Applying the results of [53] (see also [54]), we can establish that the empirical eigenvalue distribution of $\Phi^{⊙}{\left(\Phi^{⊙}\right)}^{T}$ still converges almost surely towards $\nu_{c}$, under the asymptotic regime $\frac{R}{N}\to c$. For continuous function f(λ)=log(1+λ/ρ), we apply Equation (A4), $\int_{R^{+}}\log\left(1+\lambda /\rho \right)\;\nu_{c}\left(d\lambda \right)$ can be expressed in terms of $t_{c}\left(-\rho \right)$ given by Equation (A3) (see e.g., [50]), we finish the proof.

## Appendix E. Proof of Result 4

We have $u\left(x\right)\overset{c\ll 1}{\approx }\frac{1}{x}+\sqrt{{\left(\frac{1}{x}+1\right)}^{2}}=\frac{2}{x}+1$ and $u\left(x\right)+\left(1-c\right)\overset{c\ll 1}{\approx }2\left(\frac{1}{x}+1\right)$, $u\left(x\right)-\left(1-c\right)\overset{c\ll 1}{\approx }\frac{2}{x}$, $u{\left(x\right)}^{2}-{\left(1-c\right)}^{2}\overset{c\ll 1}{\approx }\frac{4}{x}\left(\frac{1}{x}+1\right).$ Using the above first-order approximations, Equation (13) is

$$\begin{array}{c} \Psi_{c\ll 1}\left(x\right)\overset{1}{\approx }c\times \frac{x}{1+x}+c\log\left(1+x\right)-c\frac{x}{1+x}=c\log\left(1+x\right). \end{array}$$

Using the above approximation and Equation (12), we obtain Result 4.

## Appendix F. Proof of Result 5

We first denote $\lambda_{1}^{\left(q\right)}\geq \lambda_{2}^{\left(q\right)}\geq \ldots \geq \lambda_{n_{q}}^{\left(q\right)}\geq \ldots \geq \lambda_{N_{q}}^{\left(q\right)}$ the eigenvalues of $\Phi^{\left(q\right)}{\left(\Phi^{\left(q\right)}\right)}^{T}$, $1\leq n_{q}\leq N_{q}$, for 1≤q≤Q. We can notice that the eigenvalues of $\Phi^{⊗}{\left(\Phi^{⊗}\right)}^{T}$ are $\lambda_{n_{1}}^{\left(1\right)}\ldots \lambda_{n_{Q}}^{\left(Q\right)}$. Moreover, in the asymptotic regime, where $M_{q}\to +\infty$, $N_{q}\to +\infty$ such that $\frac{M_{q}}{N_{q}}\to c_{q}$, $0<c_{q}<1$, for all 1≤q≤Q, we have that $\lambda_{n_{q}}^{\left(q\right)}=0$ if $M_{q}+1\leq n_{q}\leq N_{q}$ and the empirical distribution of the eigenvalues ${\left(\lambda_{n_{q}}^{\left(q\right)}\right)}_{1\leq n_{q}\leq M_{q}}$ behaves as Marchenko-Pastur distributions $\nu_{c_{q}}$ of parameters $\left(c_{q},1\right)$. Recalling that $M=M_{1}\ldots M_{Q}$, $N=N_{1}\ldots N_{Q}$, we obtain immediately that

$$\begin{array}{cc} \frac{1}{N}\log\det\left(\mathrm{SNR}\times \Phi^{⊗}{\left(\Phi^{⊗}\right)}^{T}+I\right) & =\frac{1}{N}\sum_{n_{1}=1}^{N_{1}}\ldots \sum_{n_{q}=1}^{N_{Q}}\log\left(\mathrm{SNR}\times \lambda_{n_{1}}^{\left(1\right)}\ldots \lambda_{n_{Q}}^{\left(Q\right)}+1\right)\\ & =\frac{M}{N}\frac{1}{M}\sum_{n_{1}=1}^{M_{1}}\ldots \sum_{n_{q}=1}^{M_{Q}}\log\left(\mathrm{SNR}\times \lambda_{n_{1}}^{\left(1\right)}\ldots \lambda_{n_{Q}}^{\left(Q\right)}+1\right) \end{array}$$

and that

$$\frac{1}{M}\sum_{n_{1}=1}^{M_{1}}\ldots \sum_{n_{q}=1}^{M_{Q}}\log\left(\mathrm{SNR}\times \lambda_{n_{1}}^{\left(1\right)}\ldots \lambda_{n_{Q}}^{\left(Q\right)}+1\right)\overset{a.s}{⟶}\int_{0}^{+\infty }\ldots \int_{0}^{+\infty }\log\left(1+\mathrm{SNR}\times \lambda_{1}\ldots \lambda_{Q}\right)d\nu_{c_{1}}\left(\lambda_{1}\right)\ldots d\nu_{c_{Q}}\left(\lambda_{Q}\right)$$

Similarly, we have that

$$\frac{1}{M}\log\det\left(\mathrm{SNR}\times \left(1-s\right)\Phi^{⊗}{\left(\Phi^{⊗}\right)}^{T}+I\right)\overset{a.s}{⟶}\int_{0}^{+\infty }\ldots \int_{0}^{+\infty }\log\left(1+\mathrm{SNR}\times \left(1-s\right)\lambda_{1}\ldots \lambda_{Q}\right)d\nu_{c_{1}}\left(\lambda_{1}\right)\ldots d\nu_{c_{Q}}\left(\lambda_{Q}\right)$$

We obtain easily Result 5.
